# A research on copyright issues impacting artists emotional states in the framework of artificial intelligence

**DOI:** 10.3389/fpsyg.2024.1409646

**Published:** 2024-08-07

**Authors:** Hüseyin Kambur, Ayhan Dolunay

**Affiliations:** ^1^Faculty of Communication, Near East University, Nicosia, Cyprus; ^2^Faculty of Communication, Grand Library, Near East University, Nicosia, Cyprus

**Keywords:** copyright, artificial intelligence, art, artworks, artists, emotional states

## Abstract

Art and artistic creation serve as a means for artists to communicate with their environment, society, and the external world. However, the protection of artistic creations, as forms of communication, is not only a right for artists but also serves as a crucial safeguard that nurtures them during the creative process. Beyond the traditional issues of copyright, the significant advancements in Artificial Intelligence (AI) in today’s digital world have introduced a new debate regarding the ownership of copyright in artistic creations generated by AI. The question arises whether copyright belongs to the AI itself or to the individuals who guide the creative process behind it. In this study, based on the concepts of art, artistic creation, and emotional states, copyright issues will be examined. Data obtained from semi-structured in-depth interviews with artists and academic experts (eight artists, two communication experts, two law experts, and eight psychology experts) in the field will be analysed through content analysis to explore their perspectives regarding the discussion on emotional states, AI, and copyrights. The research highlights the variability of emotional states and their significant effects on individuals. Addressing the increasing trend of copyright issues, particularly within the framework of digitalization and inadequate legal regulations, it was found that artists’ emotional states are negatively impacted by these problems. This negative influence can adversely affect artists’ creativity and desire to produce. On the other hand, it was also identified that in artworks produced especially through AI, if artists’ rights are not protected, there is a possibility of negative emotional states arising. In conclusion, suggestions are as follows: Emphasising the importance of awareness-raising educational activities nationally and internationally, national copyright law (in Northern Cyprus) needs to be revised to protect traditional copyright and be expanded to include digital copyright, especially for works produced through AI. On an international level, emphasising the need to revise international agreements to include regulations for works produced through AI or to create a new agreement based on the importance of this issue.

## 1 Introduction

Exploring the question of what art is has been a challenging process throughout history, with philosophers seeking answers from ancient times to the present day. The quest for an artistic response to existential theories constitutes the fundamental reason for this pursuit. Plato, particularly, sought to answer the question of art by assuming a speculative approach, considering it as a reflection of entities (Barasch, 2013). Aristotle, on the other hand, presented a similar notion in a different manner, defining art as imitation (Erinç, 1988).

Regardless of the theoretical explanation of art, artworks and artists should be accepted as a whole. Copyright laws are crucial in protecting both the artwork and the artist. Idealist thinkers argue that artworks should serve artistic thought rather than aiming to make money. This is why they may not even consider populist art genres as genuine art (Balkır, 2020).

While many scholars and thinkers agree that this mindset may hinder the development of art, they often remain silent when it comes to making a profit through art (Haiven, 2015). On the other hand, despite the emotional importance of art in human life, evaluating it in a material sense is challenging. Art holds significant importance as a means of emotional expression and communication for humanity, and it is also of critical importance for artists. This is because creating artworks serves as both a means of nourishing their souls, so to speak, and as a fundamental mechanism for sustaining their livelihoods. At this point, it may be difficult to assess art’s emotional significance in a monetary context, it is natural for artists to pursue their profession not only for the sake of art but also to earn a living (Balkır, 2020).

At this point, obtaining copyright for their ideas and/or artworks is crucial for artists to be able to generate income (Akdoğan, 2001). Copyright is extremely important for artists who produce artworks. Copyright is necessary for artists to protect their rights, ideas, and earnings both nationally and internationally. In order for an artist to claim rights over their own work, they must own the copyright to the work, enabling them to prevent intellectual and artistic theft in this way (Akipek and Dardağan, 2001).

Within this framework, copyright ownership on artworks is considered highly important for artists. In this context, it can be argued that copyright infringements may lead to negative emotional states in artists, and these negative emotional states may negatively impact creativity and productivity like a domino effect. While this situation holds true even in traditional copyright approaches, in today’s digital age, the ownership of copyright for artworks produced by AI is a significant topic of debate.

In this regard, Gillotte (2020) has addressed the issue of copyright ownership in artworks created with AI in today’s digital age with the following statement: “Assuming that an AI-generated work is copyrightable, we turn to the question of who owns that copyright.” Additionally, the US Copyright Office and many academics have argued that copyright cannot belong to AI due to its lack of legal personality (Gillotte, 2020). However, some authors also suggest that copyright could be shared between the artist who contributed to the creation of the work and the AI (Darvishi et al., 2022).

At this point, the central question or problem of this issue revolves around whether the copyright of relevant works belongs to AI, the individual guiding it, or both. Hence, this issue needs to be carefully addressed. Because, the emotional states of artists significantly influence their productivity and creativity (Flaherty, 2011). An approach that may be considered as a violation of rights by artists could lead to negative emotional states and the aforementioned effects for their creativity and productivity too.

## 2 Basic concepts

### 2.1 Artificial intelligence

Initially introduced in academic articles, the concept of AI (See McCulloch and Pitts, 1943; Turing, 1950) gained prominence with the Dartmouth Conference (McCarthy et al., 2006). The ability of AI to solve intricate problems was acknowledged through the programme “Logic Theory” (Newell and Simon, 1956).

In the subsequent years, criticism arose regarding the limited capabilities of AI, demonstrating that it was not yet on par with human intelligence (See Dreyfus, 1972). However, with advancing and evolving technology, numerous studies addressed the progressing abilities of AI and its transformation (LeCun et al., 2015; Russell and Norvig, 2022).

The concept of AI can be defined as a system with abilities similar to human intelligence, fundamentally capable of performing tasks related to computer structures (Russell and Norvig, 2022). Comprising an interdisciplinary whole, AI possesses critical skills such as deep problem-solving, learning, and decision-making (Nilsson, 2010; Dolunay, 2024).

AI, which can generally be categorised into weak and strong AI, exhibits capabilities comparable to human intelligence in certain specified aspects for weak AI, whereas strong AI is closer to general intelligence levels (Kurzweil, 2005).

Having a vast application scope, AI manifests its impact in various fields such as the functioning of automatic systems, content analysis, healthcare, education, communication, etc. (Bengio, 2021). The examination of AI’s usage in the mentioned fields is indeed important; however, the focus of this study will be on its application in artistic production within the given context. Prior to delving into this subject, it would be pertinent to briefly define the concepts of art, artist, and artwork.

### 2.2 Art, artist and artwork

Art can be defined as a process resulting from the combination of individuals’ productivity, imagination, and aesthetic perception, culminating in a unified whole and subsequently expressed outwardly. The concept of art, with its wide boundaries, not only provides aesthetic and beauty satisfaction but also serves as a powerful tool for social critique, cultural expression, and the transmission of personal emotions and thoughts (Turgut, 1991). The broadness of the concept of art is due to its many subfields. Art encompasses numerous branches such as painting, music, theatre, sculpture, dance, literature, etc. (Adajian, 2022).

According to Adorno (1997), art possesses an autonomous structure, leading to profound effects both individually and socially. In this context, as stated, art shapes the identities, histories, and cultural values of both individuals and societies. This powerful impact of art is manifested through artworks. It should be noted that the concept of an artwork, an inseparable part of art, is the tangible output of the artists’ creative processes, materialising as a product or performance with aesthetic values.

Benjamin (2008) argues that the uniqueness and aura of an ‘artwork’ are of paramount importance. In this regard, artworks must possess a unique existence and historical context. In other words, these characteristics are fundamental elements that construct the originality and historical value of the work.

The unique and historical nature of artworks, as described, transforms them from mere aesthetic objects into tools of cultural memory and social critique (Shiner, 2001). The individual and cultural elements presented by the artist during the creative process play a significant role in the interpretation and evaluation of the work. Thus, the necessity of emphasising the importance of the artist becomes apparent. Therefore, when addressing the concepts of art and artwork, it is also essential to define the concept of the artist, who is the creator of this entire process (Adajian, 2022).

‘Artist’ can be defined as an individual who produces and/or performs works of art. However, this general definition as ‘productor and/or performer’ is insufficient for defining this term. It is crucial to emphasise that the artwork expresses the emotions, thoughts, and worldview too of the individual who performs and/or creates it. Moreover, the artist not only reflects personal emotions, thoughts, and worldview but also produces or performs works that embody the impact of social issues and cultural matters too (Guoa and Guib, 2021).

The creativity and technical skills of artists nourish the originality and depth of their works. Thus, artists can powerfully reflect both their inner world and at the same time, the external world, offering a unique experience to their audience. It must be reiterated, due to its significance, that the works of artists are not merely aesthetic products but also serve as a means of communication, social critique, and cultural expression. In this context, the role of the artist extends beyond being merely a creator-performer; they can also be considered critics and cultural transmitters too (Guoa and Guib, 2021).

### 2.3 AI and art

The discussion above encompasses the concepts of AI and, specifically, art. In the context of the research subject, it is pertinent to delve into the relationship between AI and art.

AI can be interpreted as a force contributing to significant transformations in the art world. These transformations extend across a broad spectrum, influencing aspects ranging from the creation of artworks to their exhibition.

Starting with the use of AI in the production of artworks, learning models such as Generative Adversarial Networks (GANs) and Recursive Neural Networks (RNNs) play a crucial role in this domain (Wang et al., 2020). These learning models facilitate the integration of AI into the process of artistic creation. For instance, the work “Memories of Passersby I” by Klingemann (2018) incorporates portraits randomly selected using GANs, serving as an example that highlights the synergy between the artist’s creativity and the productivity of AI.

As emphasised, the relationship between AI and art not only extends to the production of artworks but also contributes to offering new aesthetic perspectives or viewpoints to artists. Algorithms capable of analysing the structure of a particular artwork and generating new pieces serve as illustrative examples in this regard (Mazzone and Elgammal, 2019).

The types of artworks that can be produced through AI, whether visual, auditory, literary, etc., are analogous to categories of artworks that can be created by individuals (See Uzun et al., 2020).

Following the brief exploration of the relationship between AI and art, it is fitting to address the concept of copyright, which constitutes a significant focal point in the study.

### 2.4 Copyrights

If we approach the concept of copyright chronologically, the art creations produced by early human communities were evaluated with a different perspective than today. In ancient times, artworks were associated with materials, and they were not valued in terms of intellectual context separately from the substances they were made of. In other words, for instance, a music piece was evaluated in conjunction with the vinyl record it was pressed onto, and a painting was considered in connection with the canvas on which it was drawn. This situation eliminated the need for artists to secure themselves financially and spiritually on an individual basis (Dolunay and Keçeci, 2017).

As time progressed, the notion that artworks were essentially intellectual structures not necessarily tied to or limited by materials began to prevail among artists. At this point, the initial step is considered to be the emergence of ‘printing privileges.’ Especially with the rise of copying and piracy markets in the mediaeval period, the concept of ‘copyright’ entered human life, marking the inception of the first examples of copyright, particularly in the field of printing (Dolunay and Keçeci, 2017).

With the proliferation of copyright, the first legal regulation was the Act Anne enacted in 1709 in England (Dolunay and Keçeci, 2017).

The aforementioned concept of copyright is extremely crucial concerning the transmission of artistic production and cultural heritage from generation to generation. It is highly important for artists to find emotional and material satisfaction when presenting their works. Moreover, in a fair environment, it is vital for artists to obtain their rights in a commercial context and be able to control unauthorised usage. In this context, the protection of copyright for artworks is perceived not only as a commercial necessity but also as highly significant for cultural advancements (Özgür, 2020).

## 3 Traditional and digital copyrights

### 3.1 Traditional regulations (national: north Cyprus and international)

Geographical timeline of copyright laws in contemporary Turkey reveals a progressive journey. In the mid-1800s, the “Hukuku Telif Nizamı” (Copyright Law) was introduced, followed by the “Hakkı Telif” (Right of Copyright) enacted by the Ottoman administration in the early 1900s. The present-day legal framework, the Copyright and Related Rights Law No. 5846, was enacted on January 1, 1952 (What is copyright?, 2014).

In the current territorial boundaries of Northern Cyprus, serious challenges in copyright enforcement have led many national artists to protect their artistic works financially and spiritually by seeking copyright under the laws of the Republic of Turkey. This is due to significant issues within the copyright system in Northern Cyprus (Violation of intellectual property rights in the TRNC violation of intellectual property rights in the TRNC, 2023).

As emphasised earlier, the ever-advancing and unstoppable pace of global technology provides immense access to artists and artworks for individuals worldwide. This phenomenon has made copyright protection more challenging, leading to widespread piracy. Copyright laws play a crucial role in legally safeguarding the intellectual creations of artists. Northern Cypriot artists face considerable difficulties in protecting their artistic ideas due to the inadequacy of the existing law (Violation of intellectual property rights in the TRNC violation of intellectual property rights in the TRNC, 2023).

On a national level, the Copyright Law in Northern Cyprus is notably insufficient. The existing law, referenced from the 1911 British law during the British colonial period, is extremely limited, represented by a 4-article Law No. 264. Additionally, there are regulations such as the Broadcasting High Board Copyright and Producer Rights Protection Law and Procedures Regulation and the Copyright Regulation (Violation of intellectual property rights in the TRNC violation of intellectual property rights in the TRNC, 2023).

Under the Copyright framework applied in Northern Cyprus, the rights granted to the copyright holder include:

1. “To produce, reproduce, perform, or publish the work.”
2. “In the case of a dramatic work, to adapt it into a novel or into a non-dramatic work.”
3. “In the case of a novel or other non-dramatic work or other artistic works, to transform it into a dramatic work by performing it generally.”
4. “In literary, dramatic, or musical works, to record, perforate with perforated waltzes, make cinematographic films, or create other devices or apparatus by which the work can be mechanically performed and represented” (Violation of intellectual property rights in the TRNC violation of intellectual property rights in the TRNC, 2023).

In the event of copyright infringement in Northern Cyprus, the copyright owner must have documentation proving ownership before initiating legal action. These cases primarily aim to prevent others from profiting from or copying and imitating creative works. It is crucial for artists to be aware that copyright protection is not indefinite, lasting for 50 years after the artist’s death (Violation of intellectual property rights in the TRNC violation of intellectual property rights in the TRNC, 2023).

Examining various countries’ national regulations, leading countries in copyright protection, such as Germany, the United Kingdom, and Spain, have comprehensive laws known as ‘Intellectual and Artistic Works Protection Laws.’ These laws protect both written and printed works as well as draft and conceptual works, highlighting the ownership of the artist (Turan, 2014).

Internationally, some crucial agreements include:

Bern Convention (1886): The oldest and most fundamental regulation governing copyright, signed in Bern, Switzerland, in 1886, providing mutual legal protection among member nations.

World Intellectual Property Organisation (WIPO) Agreement: Aims to universally regulate intellectual and artistic works’ protection on a global scale, similar to the Bern Convention (Gin, 2004).

Rome Convention (1961): Primarily focuses on protecting sound recordings internationally.

TRIPS Agreement (1994): Aims to unite companies and rights holders for universal protection of intellectual property, specifically in trademark matters.

The present-day evolution of the artist and artwork, undergoing rapid change, coupled with the impact of social media, digitization, and the emergence of AI, has transformed copyright issues into a more complex and globalised landscape (Kaynak and Koç, 2015).

### 3.2 Traditional to digital

Traditional copyright laws have long been an effective tool for protecting the ideas and works of artists and ensuring their rights are respected. However, with the development of digital structures, social media, and art platforms, adapting copyright to digital versions has become inevitable (Litman, 2020).

Digitalised copyright, tailored to the digital environment, is considered to be more solution-oriented compared to traditional copyright methods, as it not only protects the rights of intellectual and artistic creators on an international scale but also provides fair and controllable conditions in areas such as equal access, rapid compensation processes, and work diversity (Litman, 2020).

In addition to digital platforms, the concept of art has evolved into a new dimension today, with professionally produced artworks created with AI. When examining artworks and ideas generated by AI, there is observed a divergence of opinions among individuals studying this field. Especially in the realm of art, the majority tends to accept the idea that AI is an object without personality and does not possess intellectual ownership. Therefore, artworks produced by AI are generally considered to be owned by the one directing it (Zorluel, 2019).

On the other hand, considering the analysis and production capabilities of today’s computers, some argue that AI can also fulfil the emotional aspect of art creation. In this regard, attributing the intellectual ownership of artworks to AI is not surprising (Zorluel, 2019).

A group that combines and evaluates the two contrasting views mentioned above argues that the usage rights of works produced with AI are equally divided between AI and the person directing it (Zorluel, 2019; Darvishi et al., 2022).

Apart from the issue of ownership of copyright in artistic productions with AI, important aspects such as how it will be protected, etc., are not yet fully regulated. In national regulations, including the United States (Isohanni, 2021), there are no regulations in this regard. This issue, due to its current relevance, is a topic of academic discussions. It is noteworthy that even the United States, often cited as an example in these academic debates for its legal regulations, does not yet have a comprehensive legal framework on this matter (Škiljić, 2021). In particular, the international regulations mentioned above are not up-to-date in the face of today’s technology. These regulations seem to be outdated, as they are based on the idea that only individuals can create works that will be protected by copyright. However, with the emergence of works produced by or with the help of AI, these regulations have become inadequate to meet current needs (Hristov, 2020).

Additionally, without straying from the focus of the topic, it is important to note that discussions regarding artworks produced through AI continue not only from a legal standpoint but also from an ethical perspective. Notably, literature includes warnings that their use in media could lead to disinformation, mass manipulation, and the proliferation of large amounts of low-quality content. However, while this is a related issue, it constitutes a separate debate. Therefore, it has been briefly addressed in this study due to its significance (Vyas, 2022).

## 4 Relationship between emotional state and artistic production

### 4.1 Emotional state

The concept of emotional state can be defined as a general evaluation of an individual’s emotional condition at a given moment (Ekman and Davidson, 1994). Emotional states are categorised as positive, negative, or neutral. Emotional states reflect individuals’ emotional experiences in the specified categories and manifest themselves physically, behaviourally, and mentally (Keltner and Gross, 1999).

Emotional states are closely linked to brain structures and functions. The limbic system, particularly structures such as the amygdala and hypothalamus, plays a crucial role in regulating emotional responses (LeDoux, 1996). Neurotransmitters, especially serotonin and dopamine, have a critical role in transmitting chemical signals that affect emotional states (Hariri and Holmes, 2006).

When considering the formation of emotional states, the complexity of genetic, environmental, and cognitive factors becomes apparent (Gross and Thompson, 2007). Determining factors include individuals’ past experiences, general personality structure, and emotional regulation strategies acquired from childhood onwards (John and Gross, 2004).

Emotional states significantly impact individuals’ quality of life. For instance, positive emotional states can enhance individuals’ motivation and strengthen overall feelings of well-being (Fredrickson, 2001). However, negative emotional states can create stress and be among the factors contributing to conditions such as anxiety and even depression (Watson and Clark, 1984).

In academic studies, it is noted that individuals’ emotional states are crucial; rapid and unjust progress goals can lead to unethical approaches, and the unethical use of AI is cited as an example of these unethical approaches (Dolunay and Temel, 2024).

On the other hand, considering the significance of emotional states in all areas of life, it can be argued that they are particularly important for artists when thinking about how emotional states are expressed. In this regard, it is pertinent to discuss the effects of emotional states on artistic production.

### 4.2 Effects on artistic production

As mentioned above, emotional states significantly impact individuals, and in this context, they also have important effects on the creativity of artists. For example, according to Csikszentmihalyi’s Flow theory, positive emotional states are believed to enhance creativity and allow for deeper connections in artistic production (Moneta and Csikszentmihalyi, 1996).

Moreover, neurological studies also explore the connection between emotional states and artistic production processes. Interactions with artworks are suggested to increase activity in the emotional regions of individuals’ brains (Kawabata and Zeki, 2004). Research on how emotional states affect brain activities during artistic production processes contributes to the biological understanding of the subject.

Art is considered to be a method for artists to express their emotional states through the creation of artworks (Garrido and Schubert, 2015). These processes also contribute to establishing emotional connections between viewers and art (Juslin and Sloboda, 2010).

Studies suggest that emotional states influence various processes in artists’ works, from composition to colour selection (Smith et al., 2015). Similarly, studies using brain imaging techniques have found that positive emotional states support activities in brain regions associated with creativity (Flaherty, 2011).

In numerous scientific studies, the effects of emotional states on artists have been investigated, yielding varied results. As mentioned above, some studies have found that positive emotional states enhance creativity among artists (Baas et al., 2008). However, another study suggests that negative emotions also have the potential to enhance creativity (Forgas and Baumeister, 2019). Furthermore, some studies have found no significant relationship between emotional states and creativity (Davis, 2009).

While the notion that positive emotions tend to support creativity is more common, it is also plausible to assume that certain negative emotions may also support creativity and productivity. This is because some challenges, among other factors, can serve as a form of reverse psychology, providing motivation. For example, sad situations may lead the creativity (Ashby and Isen, 1999). However, the assumption that emotional states are completely unrelated to creativity is, in our view, not universally valid. Nevertheless, these differing results underscore the importance of further research and investigation into the subject.

When considering the overall consensus of consistent views, it is concluded that positive emotional states support the creativity and productivity of artists, while negative emotional states may have the opposite effect.

In this context, concerning the crucial issue of copyright for artists, if their rights are violated or they perceive that their rights have been violated, it can be assumed that their emotional states will be negatively affected. This, in turn, could undermine their creativity and motivation to create.

To emphasise the multifaceted and contemporary nature of the issue, it is noteworthy that various aspects, such as the emotional perspectives of artists towards their self-produced musical works compared to those produced with AI assistance, are also subjects of debate. Research highlighted in the literature (See Vikström and Von-Bonsdorff, 2022) provides current and intriguing insights in this direction. These studies suggest that the emotional states of artists might be negatively impacted by the notion that individuals they perceive as less talented could produce similar works with the aid of artificial intelligence (Anantrasirichai and Bull, 2022; Vikström and Von-Bonsdorff, 2022). Given that the topic is discussed from many angles, this study specifically explores the emotional states of artists regarding the issue of copyright ownership in AI-generated artworks, addressing an aspect that, in our opinion, could have significant implications for creativity and productivity.

## 5 Research

### 5.1 Method

This study, particularly focusing on the negative impacts of copyright issues on the emotional states of artists, aims to identify and propose solutions to the problems that may arise or have arisen. In this context, considering both national (Northern Cyprus) and international regulations, the emergence of copyright ownership issues in the context of the development of AI in the current digital age becomes crucial regarding the emotional states of artists. As mentioned earlier, negative emotional states can undermine creativity and artistic production, making the investigation of this issue even more significant. In the research section of this study, focus group interviews were conducted with artists and semi-structured in-depth interviews were conducted with academic experts in the field.

A focus group interview is defined as a discussion and interview conducted between a small group and a leader, utilising group dynamics to gather detailed and comprehensive information and generate ideas (Bowling, 2002; Çokluk et al., 2011). On the other hand, the primary concern in a focus group interview is to conduct a carefully planned discussion in an environment where participants can comfortably express their views and opinions (Krueger and Casey, 2000). The aim at this point is to obtain in-depth and comprehensive qualitative information on individuals’ perspectives, interests, experiences, tendencies, thoughts, perceptions, feelings, attitudes, and habits regarding a specified topic. Focus group interviews typically consist of 8–12 participants (Kitzinger, 1994, 1995; Bowling, 2002; Çokluk et al., 2011).

Interviews are commonly used as a professional technique or auxiliary tool in various research fields within social sciences, including journalism, law, and medicine (Kahn, 1983; Tekin, 2006: 101). Used frequently as a data collection technique in qualitative research, interviews provide an opportunity for the participants to express themselves directly, allowing the researcher to conduct comprehensive observations about the interviewees (McCracken, 1998: 9; Tekin, 2006: 102). The interview, by asking questions covering all dimensions of the research topic, enables the collection of detailed answers, facilitating one-on-one information gathering (Johnson, 2002: 106; Tekin, 2006: 102).

Interviews can be categorised into three subtypes: unstructured, semi-structured, and structured (Punch, 2005: 166; Tekin, 2006: 104). Semi-structured interviews involve pre-prepared questions but allow the researcher to direct additional questions based on the course of the interview.

In this study, focus group interviews and semi-structured in-depth interview techniques were chosen for its relevance to the topic.

### 5.2 Sampling

In the context of research, the term “population” refers to the entirety of individuals with similar characteristics, while smaller groups that can be selected from within the population and have the power to represent it are referred to as “samples” (Yıldırım and Şimşek, 2018). The snowball sampling technique was employed to identify both the focus group (artists) and the groups interviewees (academic experts).

The snowball sampling technique is a method that involves selecting a reference person related to the subject of the study and reaching other individuals through recommendations. This method is iterative, and participants guide researchers, contributing to the growth of the sample. Therefore, it is known as the “snowball effect” (Biernacki and Waldorf, 1981).

The interview group identified through snowball sampling consists of eight artists (two painters, two graphic artists, two musicians, two photographers) and eight psychology experts, also two communication experts and two legal experts too.

Artists were included in the sample due to their focus on the subject. On the other hand, academic experts in communication and law were included in the sample because copyright is an interdisciplinary subject from both legal and communicative perspectives (Mengüşoğlu, 2015). On the other hand, the psychology experts were included in the sample for understanding the emotional states term, relationship between copyrights and artists emotions, emotional effects of copyrights issues on artists creativity.

While there was no geographical limitation in the context of the universality of the subject in snowball sampling, certain criteria were sought for proposing names to be included in the sample. Among these criteria were the involvement of artists in one of the fields of painting, graphics, music, or photography to create a diverse artistic perspective in the sample, having at least 5 years of professional experience to benefit from the experiences of experienced individuals, and for artists to have academic studies in the relevant fields (being academics). Similarly, for communication and legal experts, the criteria included having at least 5 years of professional experience to benefit from the experiences of experienced individuals and holding a doctoral degree in the fields of communication and law to bring an academic perspective to the subject.

On the other hand, as mentioned above, since creating a comfortable environment and careful planning are essential in focus group interviews and interviews in general, to ensure participants can openly express their opinions, the interviews were conducted online to minimise the effects of external stimuli. This approach aimed to enable focus group participants to express their views freely in their natural environments. However, considering the possibility that participants, even in their natural settings and experienced in their fields, might provide biased responses due to an overly individual perspective, academic artist-scholars with professional academic experience and titles were included in the focus group. This inclusion aimed to obtain more objective, ethical, and unbiased responses from an academic perspective.

Similarly, the primary purpose of selecting field experts (psychologists, legal experts, and communication specialists) from among academics with professional experience and titles at universities was to maintain objectivity and academic rigour. As focus group interviews typically consist of 8–12 participants, it was deemed sufficient to conduct a focus group interview with eight artist-scholars relevant to the subject. Since the focus group comprised eight individuals and given the equal importance of the psychologists in the context of the study—as professional evaluators—interviews were conducted with eight academic psychologists. While the groups of artists and psychologists each consisted of eight members, the groups of legal scholars and communication scholars consisted of only two members each. The reasoning behind this was to avoid deviating from the main focus of the study while still supporting it from legal and communicative perspectives, as copyright issues encompass both areas.

Details regarding the analysis of the data obtained from focus group interviews and semi-structured in-depth interviews are provided below.

### 5.3 Analyses

The data obtained in the research were evaluated using the content analysis method. Content analysis is the neutral, systematic, and quantitative description of the content resulting from communication (Berelson, 1952: 17; Koçak and Arun, 2006: 22). Another definition characterises content analysis as a research technique used to derive repeatable and valid results from data (Krippendorff, 1986: 25).

According to yet another definition, content analysis is a research technique where valid interpretations extracted from the text are articulated through successive processes (Weber, 1989: 5; Koçak and Arun, 2006: 22).

In line with the nature of the study, sometimes detailed coding is required, while at other times, comprehensive coding may not be necessary (Karataş, 2017: 80; Yıldırım and Şimşek, 2018: 233). Therefore, due to the ease of evaluating the data obtained through the conducted interviews in the context of the study, a more intricate coding and categorisation theme was not deemed necessary.

In this context, the data obtained from focus group and semi-structured in-depth interviews with sampling were themed and coded as follow:

In the analyses under the specified themes and codes (Table 1), 20% of the direct opinions of the interview group were included. The names of the participants are given in codes as A1, A2, A3, etc. (for Artists), P1, P2, P3, etc. (for Psychology Experts), L1, L2 (for Law Experts) and C1, C2 (for Communication Experts).

**Table 1:** Themes and codes.

	Themes				
	Copyright: importance, general status	Emotional states, and creativity	Regulations of copyright (national and international)	The impact of copyright infringements on artists’ emotional states and copyright ownership in AI-generated content production	Inadequacy of regulations in the context of copyright issues and proposed solutions
Codes	Importance of Copyright	Emotional States	National (North Cyprus) Regulations	The Relationship between Copyright Issues and Emotional States	Inadequacy of Regulations of Copyrights
	General Status	Emotional States and Creativity	International Regulations	Copyright Ownership in AI-Generated Content Production	Proposed Solutions
				The Emotional Impact on Artists When AI Holds Exclusive Copyright	

### 5.4 The importance and general status of copyright

#### 5.4.1 Importance of copyright

The matter of copyright is considered a fundamental value in European countries and holds significant importance within the legal domain. Particularly, due to the rapid development of digital platforms in recent times, copyright has been subject to serious infringements, necessitating concrete and ultimate legal remedies through robust legislation and judiciary, as emphasised by Eren (2019). Within this regards, it is believed that copyright laws lacking strong and solid foundations lead to serious problems and infringements within the context of artists.

The interview group (artists, communication experts and law experts), entirely in line with the literature, highlighted the importance of copyright and expressed that artists’ rights over their works are protected within the framework of copyright:

A6: *“Many globally recognized artists attach great importance to the copyright of their artworks, both in terms of financial and spiritual gains.”*

L2: *“From a legal perspective, copyright laws are the most effective regulations that protect artists internationally in terms of profit and ownership on global platforms.”*

#### 5.4.2 General status

Copyright laws constitute a legal framework related to the rights granted to the creators of creative works. Broadly defined, copyright entails the producer’s exclusive rights over a work of art, including but not limited to copying, distribution, profit generation, and transfer to others. While national copyright laws may vary, international copyright agreements uphold fundamental principles.

In alignment with this perspective, the interview group (artists, communication experts and law experts) also asserted that copyright, in the context of its general status, relies on a legal infrastructure:

C1: *“...laws and legal authorities should be in place to prevent the infringement of copyright.”*

A2: *“...taking Turkey, our closest geographical neighbour, as an example, copyright is protected under the law, just as it is worldwide, through the Intellectual and Artistic Works Act.”*

### 5.5 Emotional states, and creativity

#### 5.5.1 Emotional states

Emotional states significantly impact individuals’ quality of life. For instance, positive emotional states can enhance individuals’ motivation and strengthen overall feelings of well-being (Fredrickson, 2001). However, negative emotional states can create stress and be among the factors contributing to conditions such as anxiety and even depression (Watson and Clark, 1984).

In the context of expertise, during the interview, only psychologists were asked about what emotions are. Psychologists characterised emotions as fundamental elements that can fluctuate and directly impact individuals.

P1: *“Emotions are processes that can* var*y and influence an individual’s mood positively or negatively.”*

P3: *“A state of feeling that an individual experiences over an extended period.”*

#### 5.5.2 Emotional states and creativity

In literature, some studies have found that positive emotional states enhance creativity among artists (Baas et al., 2008). Another perspective suggests that in some cases, emotional states like sadness may trigger a reverse psychology effect, influencing creativity (Ashby and Isen, 1999).

*P2: “Emotional states are directly linked to creativity. Positive emotional states enhance creativity and productivity, whereas negative emotional states can hinder them. However, in some cases, for instance, an artist in a melancholic state may still produce a creative work. Yet, generally, positive emotional states will more strongly support creativity and productivity.”*

*P4: “Emotional state influences creativity and productivity. Generally, positive emotional states will have a positive impact on generating and creativity.”*

### 5.6 Regulations of copyright (national and international)

#### 5.6.1 National (north Cyprus) regulations

In the context of national copyright matters, the Copyright Law in Northern Cyprus is notably inadequate, mirroring the deficiency observed in many laws within the region. Furthermore, it is worth noting that Northern Cyprus has not independently formulated the law currently in effect within its borders. Influenced by the island’s historical hosting of various civilizations and the dominance of the United Kingdom before Turkish sovereignty, the existing Copyright Law, Chapter 264, consisting of only four articles, refers to the 1911 law of the UK, yet remains highly insufficient (Violation of intellectual property rights in the TRNC violation of intellectual property rights in the TRNC, 2023).

The study group (artists, communication experts and law experts), unanimously, expressed that the existing Copyright Regulations at the national level are inadequate:

A3: *“I am aware that the rights of use for works of art are not sufficiently protected in Cyprus. We have encountered several difficulties, especially in terms of commercial aspects of artistic works.”*

L1: *“Copyrights are not protected in our country.”*

#### 5.6.2 International regulations

Universal copyright regulations that are deemed significant include:

Berne Convention (1886): Historically, this is the most rooted and ancient regulation within the framework of copyright. Signed in the city of Bern, Switzerland, in 1886, the convention establishes fundamental approaches and provides mutual legal protection among member nations. It aims to safeguard the rights of artistic works concerning ideas and ownership (Berne Convention, 1886).

World Intellectual Property Organisation (WIPO) Agreement: The WIPO agreement is a global regulation that addresses works in terms of both intellectual and ownership aspects. Similar to the Berne Convention mentioned above, it aims to protect international intellectual and artistic works.

Rome Convention (1961): Primarily focusing on sound recordings, the Rome Convention defends the rights of printed sound works on an international scale.

TRIPS Agreement (1994): The TRIPS (Trade-Related Aspects of Intellectual Property Rights) Agreement aims to unite companies and intellectual property holders in protecting copyright, particularly in the context of branding, for those who are members.

Within the interview group, 50% of the respondents referred to the aforementioned agreements in response to the relevant question:

L1: *“Copyrights are internationally safeguarded by agreements. For example, the Rome Convention, the Berne Convention are among the important regulations....”*

### 5.7 The impact of copyright infringements on artists’ emotional states and copyright ownership in AI-generated content production

#### 5.7.1 The relationship between copyright issues and emotional states

Art is considered a method of expressing the emotional states of artists, the process of producing art, and the emotions of the artists themselves (Garrido and Schubert, 2015). These processes also contribute to establishing emotional connections between viewers and art (Juslin and Sloboda, 2010).

Studies suggest that emotional states influence various aspects of artists’ works, from composition to colour selection (Smith et al., 2015). Furthermore, research utilising brain imaging techniques has identified that positive emotional states support activities in brain regions associated with creativity (Flaherty, 2011).

In the context of emotional states, psychologists with their expertise on copyright issues have stated that the emotions of artists whose copyrights are violated will be affected.

P5: *“The emotional states of artists whose copyrights are violated will be adversely affected.”*

P6: *“Artists whose rights or directly copyrights are violated will experience adverse effects both financially and emotionally, impacting their motivation to create and consequently, their creativity.”*

#### 5.7.2 Copyright ownership in AI-generated content production

In the literature, there is an ongoing debate regarding the ownership of copyright for artworks generated by AI. According to the first perspective, copyright ownership should belong to the AI, while the second perspective argues that it should be attributed to the individual who directs and contributes intellectual effort to the AI. The third perspective suggests that copyright should be jointly owned by both the AI and the individual.

The interview group (artists, communication experts and law experts), approximately ≌83%, expressed the view that copyright should be equally shared between AI and the individual:

A5: *“Since a work of art is created through the collaborative efforts of both the artist and the AI program, the copyright ownership should be divided equally.”*

A8: *“In a collaborative work where both parties contribute, the distribution of copyright should be equal.”*

On the other hand, approximately ≌8% of the interview group stated that only the individual should hold the copyright, while another ≌8% argued for the sole copyright ownership of the AI.

#### 5.7.3 The emotional impact on artists when AI holds exclusive copyright

As mentioned earlier, the emotional states of artists significantly influence their productivity and creativity (Flaherty, 2011). If artists cannot claim copyright for a work they have put effort into producing, it is possible that they may be emotionally affected negatively.

The questions at this point were directed towards artists and psychology experts in their respective fields to understand emotional states. Approximately ≌87.5% of the interview group expressed that artists would not be satisfied if copyright for works produced using AI belonged solely to the AI. They emphasised that such a situation could lead to negative emotional and creative consequences for artists:

A1: *“I believe that the inability of artists to claim rights for artworks produced from their own ideas will lead to reluctance in the production process.”*

P7: *“The absence of copyright on a work that an artist has put effort into will negatively impact their emotional state, reducing their desire to create.”*

On the other hand, around ≌12.5% expressed the opinion that the emotional states of artists should not be affected if only AI holds the copyright.

### 5.8 Inadequacy of regulations in the context of copyright issues and proposed solutions

#### 5.8.1 Inadequacy of regulations of copyrights

In North Cyprus, national copyright laws and regulations are known to be inadequate. Even if copyright is obtained for works within the framework of these laws, the protection of this copyright will not be universally recognised on an international platform since the country is not acknowledged (See Tamçelik, 2013; Dolunay and Kasap, 2020). In this regard, making an international agreement and raising awareness among artwork owners through state-supported programmes will play a crucial role in preventing unauthorised developments (Dolunay and Keçeci, 2017).

The interview group, based on the conducted interviews, unanimously expressed that the existing copyright law in effect in Northern Cyprus is insufficient:

A7: *“In my opinion, our artists’ artworks are not adequately protected. In fact, in many cases, artists find themselves having to personally safeguard their works.”*

L2: *“Due to legal gaps, our artists are in a very disadvantaged situation on a national level. They often fall victim to intellectual property theft, and unfortunately, there is no well-organised protective law they can rely on to assert their rights.”*

#### 5.8.2 Proposed solutions

Intellectual property regulations have been established based on the notion that only individuals can create works eligible for copyright. However, as indicated, these regulations have become inadequate in addressing current needs, particularly concerning works generated by or through AI and the associated copyright issues (Hristov, 2020).

The interview group expressed similar views, highlighting the insufficiency of international regulations in meeting contemporary needs, especially in the copyright processes of artworks created with AI. Consequently, they proposed solutions in this regard.

The interview group provided various recommendations to prevent or address copyright issues. Each participant responded with multiple suggestions, broadly categorised under two main headings:

In the context of the importance of copyright, proposing widespread educational activities to increase respect for and awareness of copyright:

A2: *“Certain institutions need to educate individuals on this matter. I believe that individuals involved in the arts, seeking copyright for their artworks, should be obligated to attend an initial training.”*

Development of national and international legal regulations related to copyright / Establishment of regulations for digital copyright and works generated through AI:

A7: *“I believe that traditional copyright laws have reached their limits, both in national and universal contexts. I am of the opinion that all copyright issues need to be resolved in the context of digital technology, ensuring easy accessibility and a lasting solution.”*

## 6 Findings and discussion

Copyright, a crucial system enabling the protection of artists’ rights over their works, is grounded in its legal status. As with any right, it is essential to respect and adhere to legal regulations concerning this right.

In the study, the interview group emphasised the importance of copyright and indicated that its status is based on a legal foundation when defining copyright. This approach aligns with the perspective in the literature, emphasising the significance of copyright within the framework of copyright law (Vaver, 2000).

Emotional state, often relying on long-term accumulation, can typically exhibit moment-to-moment fluctuations. It is a concept used to express individuals’ positive, negative, or neutral feelings (Fredrickson, 2001). In this study, it was determined that emotional states could indeed be positive, negative, or neutral, consistent with this notion in the literature.

In the literature, highlights an association between emotional states and creativity, suggesting that positive emotional states generally positively influence creativity and the desire to produce (See Baas et al., 2008). Consistent with this approach, this study found that emotional states are linked to productivity and creativity, and the positive emotional states increase creativity and productivity.

On the other hand, the common belief that negative emotional states adversely affect creativity and productivity was considered in this study and presented as a finding too. However, there is also an approach in the literature suggesting that some negative emotional states (such as sadness), through a reverse psychology effect, may support creativity and productivity (Ashby and Isen, 1999).

It is emphasised once again that while positive emotional states positively influence creativity and productivity, negative emotional states generally have an adverse effect on aspects of creativity and productivity for artists. However, with the perspective mentioned above, it was also found in this study’s research findings that certain negative emotional states, such as sadness, might positively trigger creativity and productivity through a reverse psychology effect (as exceptions).

The regulation of copyright, stemming from its legal foundation, is made possible through various legal arrangements. In the context of this study, the issue is regulated nationally (in Northern Cyprus) by the Copyright Law Chapter 264. On the international front, crucial agreements such as the Berne Convention (1886), the World Intellectual Property Organisation (WIPO) Agreement, the Rome Convention (1961), and the TRIPS Agreement (1994) regulate the issue.

In the study, the interview group also mentioned the laws and agreements mentioned above concerning national (Northern Cyprus) and international regulations.

Furthermore, considering the importance and legal regulations, the connection between copyright and the emotional states of artists is crucial. The concept of emotional states can be defined as an individual’s overall assessment of their emotional condition at a given moment (Ekman and Davidson, 1994). Emotional states can be categorised as positive, negative, or neutral. Positive emotional states can enhance individuals’ motivation and overall feelings of well-being (Fredrickson, 2001). On the other hand, negative emotional states can create stress and contribute to conditions such as anxiety and even depression (Watson and Clark, 1984).

In the study, it was found that if artists encounter copyright issues, as expected, there will be negative effects on their emotional states. Negative emotional states can not only affect artists’ overall well-being but also have a negative impact on their desire to create and their creativity.

When examining the process in the context of AI, it was observed that the issue of copyright ownership arises concerning works produced through AI. In the literature, there are three different approaches to the copyright of artworks produced through AI: 1. ownership to AI, 2. ownership to the individual or individuals directing and contributing to the work, 3. joint ownership shared by both (Darvishi et al., 2022).

All these views lead to separate discussions. The suggestion that AI does not have a separate personality and only the individual should have copyright ownership, the proposal for joint ownership, or the idea that AI, as the creator, should have copyright are suggestions that need to be examined in detail.

In the study, the interview group mostly stated that the individual’s intellectual effort and guidance are crucial. However, if the production is carried out with the help of AI, they emphasised that copyright should be in partnership between the individual and AI.

On the other hand, regarding the views mentioned above, the study found that the copyright belonging to AI would negatively affect the emotional states of artists. This situation could negatively impact artists’ desires to create and their creativity.

In a national context (Northern Cyprus), the existing copyright law consisting of four articles was found to be outdated, not only regarding digital copyright but even traditional copyright. It leaves artists without basic protection.

On an international scale, although there are international agreements regarding traditional copyright, it was stated that new agreements are needed, particularly addressing digital copyright and the copyright of works produced through AI.

This assertion is consistent with the claim in the literature that national and international regulations are insufficient, especially in terms of the copyright of artworks produced through AI (Hristov, 2020).

In the study, the interview group proposed solutions for preventing or resolving copyright issues:

- Emphasising the importance of awareness-raising educational activities nationally and internationally.
- Suggesting that the national copyright law (in Northern Cyprus) needs to be revised to protect traditional copyright and be expanded to include digital copyright, especially for works produced through AI.
- On an international level, emphasising the need to revise international agreements to include regulations for works produced through AI or to create a new agreement based on the importance of this issue.

## 7 Instead of conclusion: copyrights, AI, emotions and future?

All rights are significant; however, considering the nourishing, developmental, and transformative potential of culture and art on individuals, society, and the world, the importance of copyright can be emphasised.

Copyright, grounded in a legal framework, is a fundamental system that safeguards artists’ rights over their works. While traditional copyright is regulated by national laws and international agreements, the contemporary digital age has introduced AI as a significant player.

Before addressing issues related to AI, it is crucial to reiterate that the violation of copyright can adversely affect the emotional states of artists, subsequently impairing their creative desires and abilities.

On the other hand, the debate over copyright ownership in works produced through AI—whether it should belong to the artist, the AI, or be shared jointly—requires careful consideration as a current and sensitive issue.

A fair approach may involve preserving ownership for the artist in the presence of their intellectual effort and unique direction, while also granting some level of rights to the AI as the creative tool. This situation may lead to various problems and debates, highlighting the need for a fair legal framework. Furthermore, legal and just considerations should be accompanied by an awareness of the emotional states that artists may experience. For instance, a scenario where only AI holds the copyright, while potentially fair legally, may negatively impact the emotional well-being of artists.

In a national context, specifically in Northern Cyprus, even traditional copyright has not been adequately regulated. In this regard, traditional copyright should be prioritised for regulation, and any legal framework should encompass artworks produced through AI, considering the demands of the contemporary era.

On the international stage, there is a clear need for the expeditious implementation of legal regulations concerning artworks produced through AI, aligning with the demands of the times. The continuously evolving technology has rendered existing international regulations inadequate.

Emphasising the importance of copyright nationally and internationally, educational activities aimed at raising awareness among artists and the general public should be organised by universities at the national level and international intellectual property organisations.

For future academic endeavours, psychologists are recommended to conduct in-depth interviews, including aspects of artists’ emotional states affected by the violation of their rights, to delve deeper into the subject. Furthermore, as highlighted in this paper, which reexamines and analyses the effects of evolving technology, it is crucial to identify and discuss the benefits and drawbacks of the digital age. In this context, it is recommended that academic research systematically investigating the use of AI in artistic works be conducted in collaboration between artists and AI experts. Additionally, given the interdisciplinary nature of the topic, it is suggested that communication, ethics, and legal experts collaborate to deeply explore the communicative, ethical, and regulatory aspects of the issue. Communication specialists should address the communicative dimensions, ethics experts should examine the ethical considerations, and legal professionals should investigate the regulatory aspects, ultimately developing concrete proposals for communicative, ethical, and legal frameworks.

## Data availability statement

The original contributions presented in the study are included in the article/supplementary material, further inquiries can be directed to the corresponding author.

## Ethics statement

The studies involving human participants were reviewed and approved by the Near East University Scientific Research Ethics Committee. The studies were conducted in accordance with the local legislation and institutional requirements. The participants provided their written informed consent to participate in this study.

## Author contributions

HK: Formal analysis, Methodology, Writing – original draft, Writing – review & editing. AD: Formal analysis, Methodology, Writing – original draft, Writing – review & editing, Conceptualization, Project administration, Supervision.
